# Cultivars and Their Developmental Phases Interact with Temperature Fluctuations to Modulate Growth, Productivity and Seed Tuber Physiology of Potatoes (*Solanum tuberosum* L.)

**DOI:** 10.3390/plants14050750

**Published:** 2025-03-01

**Authors:** Morgan D. Southern, Mohan G. N. Kumar, Jacob M. Blauer

**Affiliations:** Department of Horticulture, Washington State University, Pullman, WA 99164-6414, USA

**Keywords:** planting time, physiological age, chronological age, accelerated aging, postharvest quality, stem number, temperature

## Abstract

In view of raising concerns of climate change, the impact of temperature on potato (*Solanum tuberosum* L.) growth and productivity was investigated by planting at different times to expose plants to natural variations in air and soil temperatures. Over two seasons with differing temperature patterns, emergence, stem and tuber numbers, tuber size distribution, yield, processing quality, and seed tuber behavior were analyzed. Postharvest, tubers from each planting were stored and replanted to assess temperature carryover effects. Generally, delayed plantings increased the average number of stems per plant (37%) but did not alter the tuber numbers per plant. Early (18 April) and mid-season (9 May) plantings produced higher yields, while late planting (30 May) reduced total yield (42%), US No. 1 yield (48%), and tuber numbers (34%). Moreover, the storage period influenced subsequent stems per plant more than the prior-year temperature conditions. Optimal productivity was achieved by planting during cooler establishment temperatures, followed by warmer tuberization and relatively cooler bulking temperatures. Diurnal temperature variations and growing degree days had minimal effects on stems per plant, whereas storage duration (chronological age) and temperature significantly impacted physiological aging. These findings help growers optimize planting times to enhance tuber storability and yield to improve end use.

## 1. Introduction

Potatoes (*Solanum tuberosum* L.) are produced globally on 20 million hectares in 150 countries, with an annual production of over 360 million tons [1,2,3]. The growth and development of the potato is modulated by the interaction of genotype with temperature and photoperiod. Although most cultivars are bred to set tubers irrespective of the photoperiod, temperature exerts profound effects on various aspects of potato growth and development such as emergence, tuberization, tuber shape, yield, and tuber maturity [4]. Previous studies carried out in greenhouses and growth chambers suggest that air temperatures above 30 °C and soil temperatures above 20 °C promote vegetative growth at the cost of tuber development [4,5,6,7,8,9]. However, studies addressing the effect of variation in day and night temperatures during the ontogeny under field-setting are limited.

Changing weather patterns, driven by global warming from increasing levels of atmospheric carbon dioxide, is raising concerns among potato producers. Temperature fluctuations and associated heat waves [10,11,12] are increasing in frequency. It is predicted that a 1 °C rise in global temperature may result in a 1.8 to 3.2 times increase in the number of extreme weather events every 10 years [13]. Compared with the mean global temperature from 1951 to 1980, the temperature in 2021 increased by 1.02 °C [14]. In 2021, the Pacific Northwest of the United States of America (PNW; specifically, the Columbia basin, a major potato producing area) suffered a ‘heat dome’ with maximum air temperatures reaching as high as 45.5 °C from the end of June to early July [15]. During this unprecedented heat wave in many areas of PNW, the maximum air temperature increased by more than 5 °C over that of previous years and this trend is expected to continue in future years [16,17]. Agronomic practices to mitigate the ill-effects of intermittent high temperature remain to be established/standardized, so growers at present are at a disadvantage to manage their crop to minimize the high temperature-induced effects on crop production. As per the predictions of International Panel on Climate Change [13], the increase in average climate temperature has the potential to cause extreme weather episodes in and around PNW in the future.

The extent of impact from high temperature depends on the genetics and the coinciding developmental phase of the crop. During the onset of tuberization, higher soil and air-temperatures encourage vine growth at the cost of tubers [4]. During tuber bulking, high temperatures promote ‘heat sprouts’ [18] and likely enhances the incidence of diseases and misshapen and/or malformed tubers [19,20,21]. Tuber shape is an important variable for visual and physical quality, and for final crop financial value. Tubers in the United States are generally graded into US No. 1s and US No. 2s (marketable yield) when sold and these categories are correlated to contract pricing, consumer acceptance, and waste estimates. Generally, both categories are considered “clean” and “firm” potatoes that are free from damage and defects, but US No. 1s are defined as “well-shaped” and US No. 2s are defined as “not seriously misshapen” [22,23]. Plants experiencing high temperature late in the production season suffer during the bulking period, which can reduce yields and result in poor shape. Studies designed to artificially increase soil temperature during the bulking period have been shown to produce smaller tubers with lower specific gravity [24], which are undesirable for processing into chips/fries. Moreover, high temperature during tuber development mitigates dormancy, reduces shelf life and accelerates physiological aging (PAGE). Such tubers are unsuitable as propagules due to their poor productivity [24].

At present, there are no cultivars directly marketed as tolerant to high temperature and attempts to develop such cultivars by breeding remains to be addressed. However, the Western Regional Potato Breeding Program (WRPBP) shares germplasm among University and USDA potato breeders. This germplasm is tested across the western United States of America under different environmental conditions (Pacific Northwest through the Southwest) and provides opportunities for selection of high temperature resistant/tolerant varieties once consistent protocols are developed. The screening and development of high-temperature resistant cultivars serves the long-term interests of the potato industry. In the meanwhile, strategies to contain/manage the ill-effects of high temperatures such as manipulation of planting time and/or depth, spacing, fertility management, and irrigation frequency merit consideration.

The current potato growing season of the Columbia basin in the PNW extends from March to October and global climate change may extend this growing period [25]. To reap the benefits of an extended growing season, cultivars tolerant/resistant to high temperature fluctuations during the growing season need to be identified. Further, with the changing environment, it is important to identify the ideal planting time for specific cultivars so that the optimal day- and night-temperatures coincide with critical crop growth stages. Thus, we examined the effects of planting time on growth, productivity, and processing quality of tubers from cultivars Shepody and Russet Norkotah. Additionally, the interaction of planting time with storage temperature and duration on the physiological age (apical dominance) of tubers was assessed.

## 2. Materials and Methods

### 2.1. Planting Material and Experimental Layout

Cultivars Russet Norkotah and Shepody were selected due to their early maturing phenotype, thus making them ideal for planting time studies. Certified G3 (generation 3) potato (*Solanum tuberosum* L.) seed tubers were obtained from a commercial seed grower in Reardon, WA. in January of 2021 (2021 crop) and October of 2021 (2022 crop) and stored at 4 °C (95% RH) for the specified period (see Table 1) prior to planting. After the specified storage period, the tubers were hand-cut into 50 to 64 g seed pieces, wound healed (3 days, 12 °C, 95% RH), and field-planted at the Washington State University (WSU) Irrigated Research and Extension Unit at Othello, WA (46°47.59200′ N. Lat., 119°02.32200′ W. Long.) with a custom 2-row planter at 20 cm depth (spaced at 25.4 cm in-row and 81.3 cm between rows, respectively). At planting, tubers used for early, mid-, and late planting had a storage period (4 °C, 95% RH) of 210, 231, and 253 days, respectively (Table 1). The experiment was laid out in a randomized complete block, split-plot design (2 cultivars × 3 planting times × 5 replications). Each field plot/replication consisted of 24 test plants and included a guard plant (cultivar Chieftain) on each end of the plot to facilitate plot separation at harvest. Plots were flanked on both sides with cultivar Umatilla Russet to promote plant competition observed in commercial production. The crop was uniformly raised across all treatments following cultural practices for fertility, integrated pest management, and irrigation specific to the Columbia basin in the PNW [26].

### 2.2. Emergence and Stem Numbers

**Figure 1:**
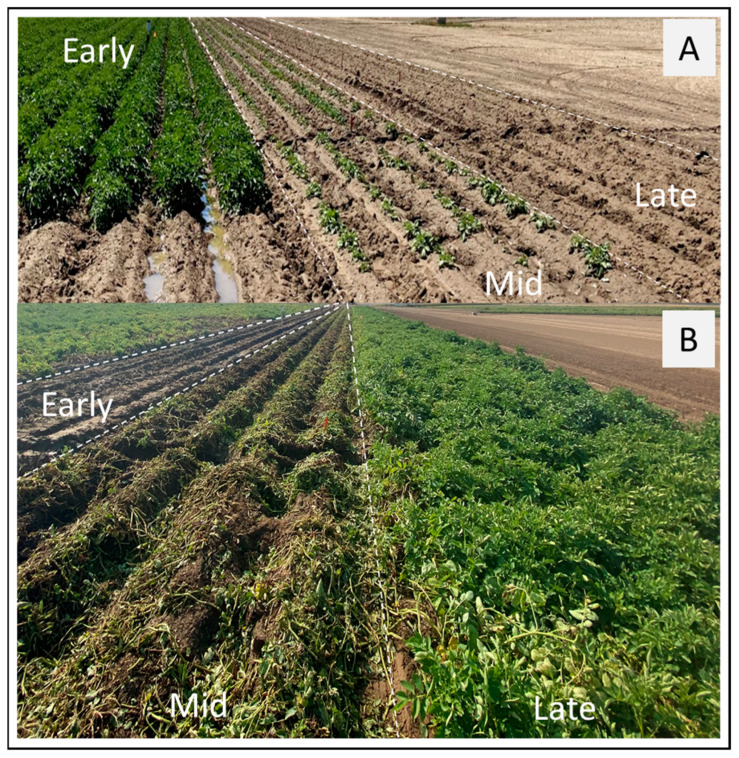
Effect of staggered planting (see Table 1 for planting dates) on sprout emergence, and crop growth and development. The experiment was laid out in a split-plot completely randomized block design with *cvs.* Russet Norkotah and Shepody (not distinguishable in the picture due to infield plot randomization). The 2022 developmental stages of early, mid-, and late plantings at 54, 33, and 11 DAP, respectively, are presented in (**A**). In contrast to mid- and late plantings, the early plantings developed a significant vine growth, and in combination with cooler spring weather of 2022, contributed to cooler soil temperatures, coinciding with tuberization. Photo (**B**) shows the crop as in (**A**) at 141 (early), 120 (mid), and 98 (late) DAP. (Note: Photo (**B**), 7 days after tuber harvest for the early planting, the day vines were mechanically removed for the mid-planting, and the actively growing vines for the late planting).

Plant emergence was monitored and recorded from 22 days after planting (DAP) until final stand establishment (approximately 60 days). Emergence was expressed as a percent of the total number of seed pieces planted. Main stems were counted at row closure (approximately 60 DAP) to calculate the average number of stems per plant. Differences in foliage based on individual planting time (2022) is presented in Figure 1A.

### 2.3. Tuber Harvest

The effect of planting time (staggered planting, as above) on tuber yields and tuber size distribution were assessed for two growing seasons (2021/2022). After 120 days, the vines were mechanically removed (vine kill) with a flail mower. Tubers were harvested 14 days after vine-kill with a Braco^®^ single-row bagger unit harvester. Differences in foliage at the time of vine removal from mid-planting (2022) can be viewed in Figure 1B. Immediately after field harvest, tubers were washed free of dirt, weighed, counted, and sorted into size categories of <113 g, 113 to 170 g, 170 to 283 g, 283 to 340 g, 340 to 396 g, and >396 g using a custom potato grader at the WSU Irrigated Research and Extension Unit at Othello, WA, USA. Total yield (MT ha^−1^) and marketable yield (US No. 1 and 2) were determined from the sorter data and by hand grading following the US grade designations developed by the USDA [23].

### 2.4. Storage, Accelerated Aging, and Replanting of Tubers

Tubers harvested from the early, mid-, and late plantings (as above) were divided into two batches and stored either at 12 °C (10 days; controls) or at 32 °C (21 days). At 12 °C and 32 °C, the aged tubers accumulated either 80 or 600 degree days (DD), respectively (based on a 4 °C base storage temperature). After the specified accelerated aging treatment (80 DD vs. 600 DD), the tubers were held at 4 °C (95% RH) until planting the following year. At planting, the tubers were hand-cut and wound-healed (as above) and planted to assess the impact of accelerated aging on percent emergence and stem counts per plant.

### 2.5. Assessment of Processing Quality

To assess the interaction of planting time and storage period, tubers were assessed for fry color as an indicator of reducing sugar level. Tuber samples were examined for fry color within a week after harvest and at 60 days of storage (4 °C, 95% RH). A total of 12 tubers from each planting time (170 to 284 g) were cut longitudinally into steak fries (9.5 mm thick, 2.9 cm wide), rinsed free of surface starch, and fried at 190 °C for 3.5 min. Light reflectance of the apical and basal ends of the fries was measured using a Photovolt 577PC reflectance meter (Photovolt Instruments, St. Louis Park, MN, USA).

### 2.6. Ancillary Data Acquisition

*Soil and air temperature:* soil and air temperatures were recorded every 15 min during the entire cropping season during the years 2021 and 2022. Soil temperature was measured at a depth of 20 cm using one of the following: ECH20 EC-5 soil moisture and temperature sensors (2021; METER Group Inc., Pullman, WA, USA), or RT-1 soil temperature sensors (2022; METER Group Inc., Pullman, WA, USA). The air temperature was measured (at approximately 1 m above ground) with a ZL6 Advanced Cloud Data Logger (2021; METER Group Inc., Pullman, WA, USA) or an ATMOS 14 temperature and humidity sensors (2022; METER Group Inc., Pullman, WA, USA). Daily average, maximum, and minimum temperatures were determined from the average of two independent field readings, one at both ends of the field test rows, for each 15 min interval reading (*n* = 192/day).

*Computation of growing degree days (GDD):* the methods of McMaster and Wilhelm [27] were used to compute growing degree days (GDD) with minor modifications. A base temperature of 7.2 °C was subtracted from the averages of two 15 min temperature recordings for soil and air (*n* = 96), then the average of the 96 reads were determined for each day. The resultant values were used to account for both the daily and the sum of the growing season GDD. Daily negative values were set to zero and no upper limit on the daily high temperature was considered for this evaluation.

*Statistical analysis:* the effects of cultivar, planting time (synonymous to storage period), postharvest degree day accumulation, and their interaction on percent emergence, average stem number per plant, tuber yield (per plant and per hectare), and tuber size distribution were analyzed statistically using analysis of variance (ANOVA) with cultivar as the main treatment interacting with planting time and degree days using JMP software (JMP 15.2 statistical discovery, LLC; San Francisco, CA, USA). Mean separation was accomplished using Fisher’s LSD. The main effect of planting time (storage time) and its interaction with cultivars to affect stem number per plant, percent emergence, and tuber size distribution, were partitioned into single-degrees-of-freedom contrasts for polynomial (linear, deviations) trends. In view of the significant variation between the years, the results from two years are presented separately.

## 3. Results

### 3.1. Air and Soil Temperatures During Crop Growth Stages

**Figure 2:**
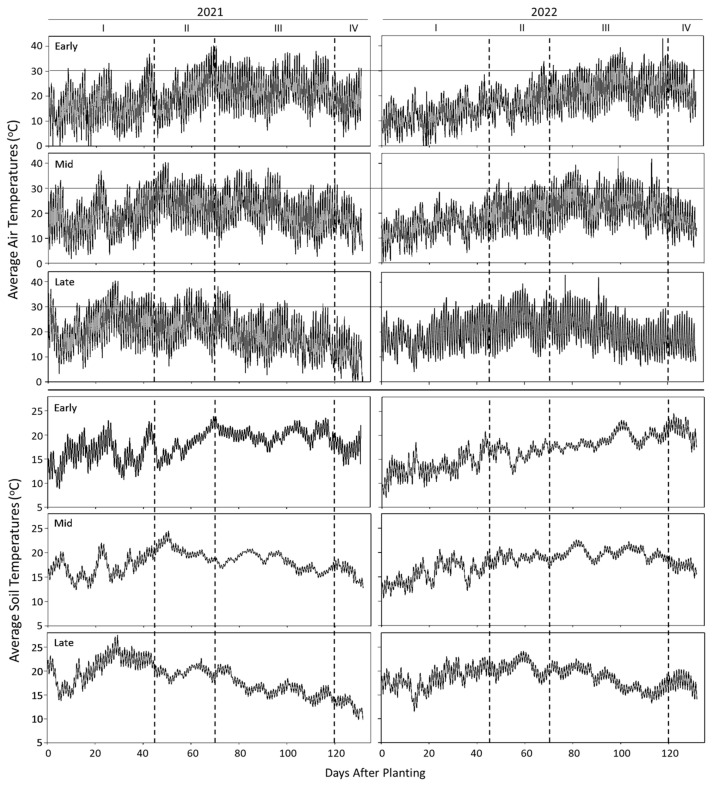
Air and soil temperatures during a 134 day cropping period (April–October) of 2021 and 2022 at Othello, WA. Note the differential exposure of developmental stages (I, II, III, and IV representing emergence, tuberization, bulking, and tuber maturity, respectively) to air and soil temperatures due to staggered planting (see Table 1 for early, mid-, and late planting dates). Temperatures were monitored daily in duplicate every 15 min for the entire cropping period. Daily averages are presented. The growing season of 2021 experienced higher soil and air temperatures than that of 2022, especially in the earlier months of the cropping season.

**Figure 3:**
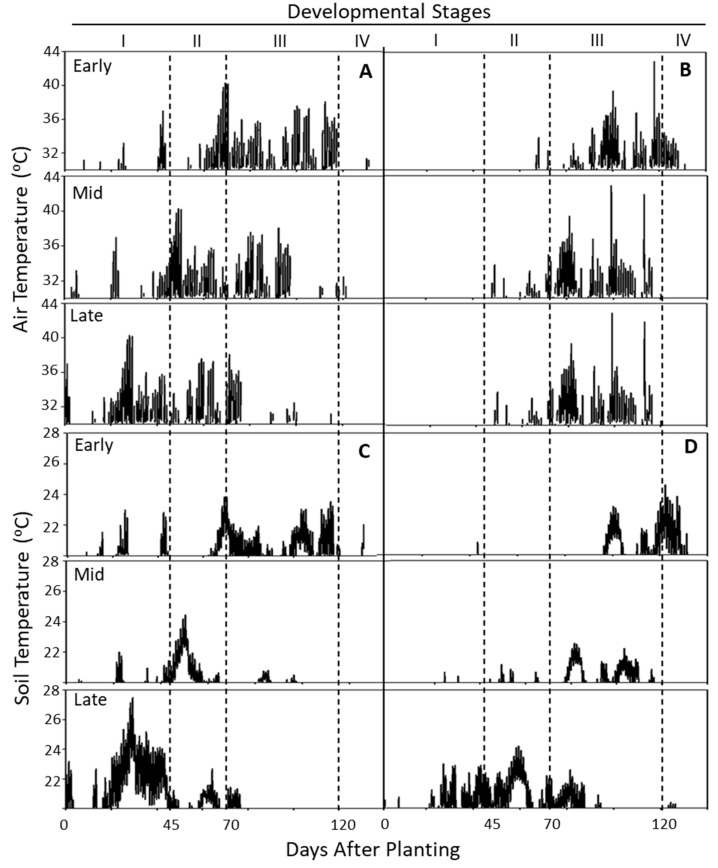
Air and soil temperatures during a 134 day cropping period (April–October) of 2021 (**A**,**C**) and 2022 (**B**,**D**) at Othello, WA. Note the differential exposure of developmental stages (I, II, III, and IV representing emergence, tuberization, bulking, and tuber maturity, respectively) to air and soil temperatures due to staggered planting. Temperatures were monitored daily in duplicate every 15 min for the entire cropping period. Air temperatures above 30 °C and soil temperatures above 20 °C (threshold for tuberization/bulking) are presented. The growing season of 2021 experienced temperatures as high as 45.5 °C (heat dome) that coincided with tuberization (stage II) of early and mid-plantings.

The soil and air temperatures during the two cropping seasons (2021/2022) were monitored continuously for the entire growing period (April–October). Daily averages of diurnal soil and air temperatures (2021 and 2022) are presented in Figure 2. In contrast to 2022, both soil and air temperatures were higher during 2021, especially during the early stages of crop growth (emergence tuberization). Temperatures above 20 °C (soil) and 30 °C (air) are known to affect tuber initiation and development, so variations in temperatures above the specified threshold levels were also considered (Figure 3). As expected, staggered planting exposed the growth stages differentially to air and soil temperatures. In contrast with 2022, the sprout emergence and tuberization stages during 2021 experienced a greater number of days with air and soil temperatures exceeding the threshold levels during different times of the day. Delayed planting was associated with increased air and soil temperatures during the emergence (Figure 3). Air temperatures during 2022 remained below the threshold levels during emergence, irrespective of planting time and generally for soil temperatures except for emergence during the late planting (Figure 3). The air temperatures during 2022 remained within the threshold levels until the crop reached tuberization and bulking. Soil temperatures of 2022 were above the threshold levels during bulking-maturity (early planting), tuberization-bulking (mid-planting) and emergence to early part of bulking (late planting). The year 2021 experienced ‘heat dome’ when the air temperature reached as high as 45.5 °C from the end of June to early July. The heat dome timing coincided with tuberization of early and mid- planting (Figure 3; stage II), and emergence of the late planting.

**Figure 4:**
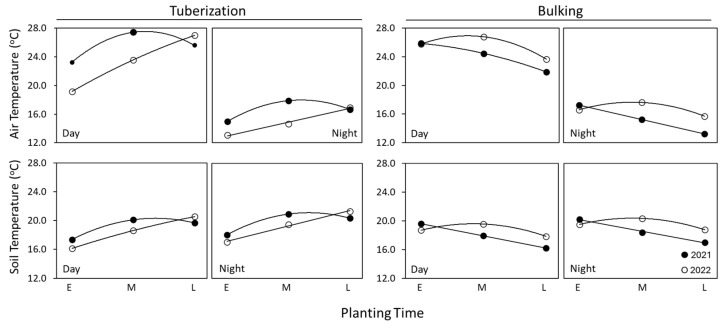
Diurnal changes in air and soil temperatures during the cropping seasons of 2021 and 2022 at Othello, WA. Note the yearly variation in the air and soil temperatures (day/night) during tuberization and bulking of early (E), mid- (M),- and late (L) plantings. Temperatures were monitored daily every 15 min during the entire crop growth period. Average values during tuberization and bulking are presented. Early and mid-plantings of 2021 experienced higher soil and air temperatures during tuberization (day and night) than that of 2022 plantings. However, during bulking, the mid and late plantings of 2021 experienced lower temperatures (air and soil) irrespective of day or night (see Table 2 for the duration and hours of day and night during potato production).

Tuberization and bulking are affected by the diurnal changes in air and soil temperatures. The number of hours of day and night during the growing season are presented in Table 2. During tuberization, plantings of 2021 (early and mid) experienced higher day and night temperatures (air and soil) than that of 2022 plantings (Figure 2 and Figure 4). Late plantings during both the years experienced similar day and night temperatures. However, during bulking, the mid- and late plantings of 2021 experienced lower temperatures (air and soil), irrespective of day or night.

### 3.2. Growing Degree Days (GDD) During Crop Growth Stages

**Figure 5:**
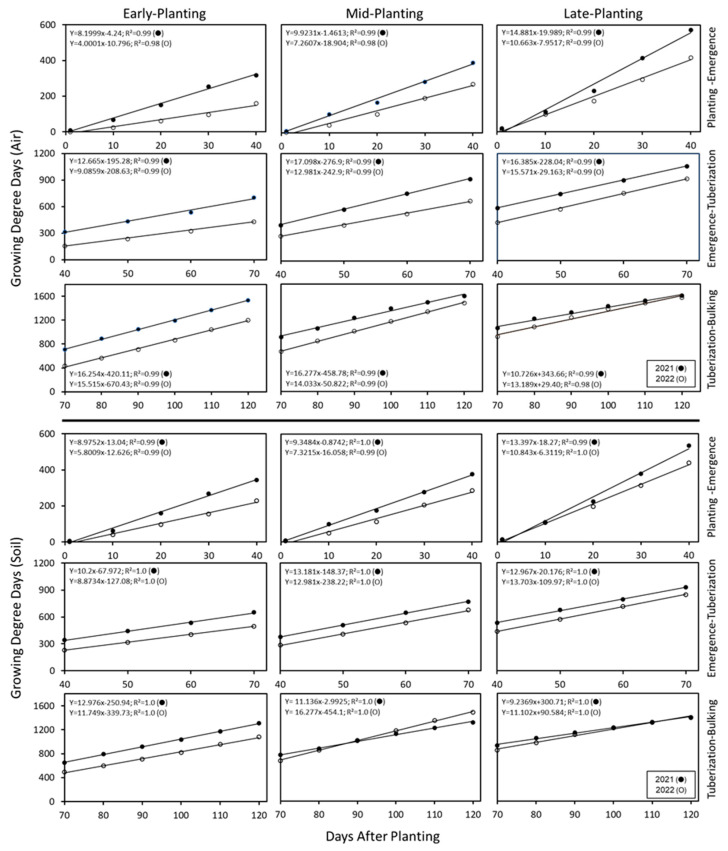
Total number of growing degree days (GDD) during the growth stages of early, mid-, and late plantings (● 2021, ○ 2022). Air and soil temperatures monitored daily every 15 min (in duplicate), and the daily averages were converted to GDDs (see materials and methods). The sum of GDDs derived from air and soil temperatures are presented. GDDs computed individually on air and soil temperatures showed similar trends. At 120 days after planting, the vines were killed, and tubers harvested after 14 days of skin-set. The GDDs increased linearly with the advancing growing period. During 2021, the number GDDs were higher, and rate of increase surpassed that of 2022, irrespective of planting time.

The number and the rate of increase in GDDs varied with planting time, developmental stage, and cropping year (Figure 5). The crop year of 2021 received a relatively greater number of total air-GDDs (air: early = 1530; mid = 1603; late = 1608) than that of 2022 (air: early = 1201; mid = 1481; late = 1575) during the 120-day cropping period. Early plantings of 2021 had the maximum benefit of air-GDDs (27% higher number of GDDs than the early plantings of 2022), a consequence of relatively warmer temperatures during the spring of 2021. Similarly, total soil-GDDs for the early plantings were higher in 2021 (21%) than 2022 (1309 vs. 1080). However, accumulation of soil-GDDs was higher by 13% in mid-plantings of 2022 than that of 2021 (1486 vs. 1318) and late plantings of 2021 and 2022 were on par (1395 vs. 1400). Regardless of the planting time or crop year, the GDDs increased linearly (R^2^ = 0.98–1.0) with advancing crop growth stages and the rate of increase was higher during 2021 except for late plantings. During the cropping period (120 days) of 2021, air-GDDs increased at a higher rate (Early: Y = 13.009x − 125.32; R^2^ = 0.98 and Mid: Y = 14.226x − 83.637; R^2^ = 99) than that of 2022 (Early: Y = 10.118x − 166.31; R^2^ = 0.94 and Mid: Y = 12.929x − 154.06; R^2^ = 0.98). The rate of accumulation of air- or soil-GDDs did not differ between late plantings of 2021 and 2022.

Within each developmental phase, the soil- and air-GDDs increased linearly (see line equations along with R^2^ values in Figure 5) with advancing days after planting. However, the rate of GDD increase varied with the cropping year and planting time. In contrast to mid- and late plating, the rate of increase in air-GDDs was higher in early plantings during ‘tuberization-bulking’ period during both years. While mid-plantings of 2021 experienced a higher rate of increase in air-GDDs during the ‘emergence tuberization’ period, in 2022, the mid-plantings had the highest rate of increase during ‘tuberization bulking’. The rate of accumulation of air-GDDs was greater during ‘emergence tuberization’ for late plantings during both years and the rate of increase did not differ significantly between the two years. The trends in soil-GDDs mirrored that of air-GDDs with minor variations. The most notable were the differences in soil-GDDs between years for mid-planting during ‘tuberization bulking’ when the mid-planting of 2022 year had a higher rate of GDD accumulation and a slightly more GDDs than the 2021 year (168 GDDs).

Interestingly, the rate of increase in air-GDDs during 2021 was consistently higher during planting emergence, irrespective of planting time when compared to the 2022 crop year (early = 105%, mid = 37% and late = 40%). Furthermore, the rate of increase in air-GDD was higher in 2021 than 2022 (by 40%) during tuber bulking for the early planting. However, for a similar growth period, the late planting recorded a 19% higher rate in air-GDDs during 2022 compared to 2021, indicating a warmer spring in 2021 and warmer late summer/early fall in 2022. Thus, these observations taken together demonstrate the yearly variation in the rate of increase in GDDs is most evident during the spring and fall of each year.

### 3.3. Sprout Emergence and Stem Numbers

As expected, planting time (early, mid-, and late) caused a shift in the onset of crop growth stages from planting to tuber maturity (Table 1). Planting time had a significant effect on the rate of emergence. Emergence was recorded at 28 days after planting (DAP; Table 3). Early plantings delayed emergence irrespective of the year or cultivar. Among the two cultivars, emergence was delayed more in Shepody. In the year 2021, the emergence of sprouts from early plantings of Russet Norkotah and Shepody were 84% and 65%, respectively. In the year 2022, the early plantings had no visible emergence for either cultivar at the same time period as 2021. Thus, early planting delayed emergence with a significant year effect. With advancing planting time (mid to late), the rate of emergence improved in both cultivars during both years, though late plantings in 2022 caused a reduction in emergence (Russet Norkotah 19%, and Shepody 14%) by 28 DAP. Planting time affected only the rate of emergence, and not the final crop-stand. Irrespective of the treatments, the final crop stand reached 100%.

**Figure 6:**
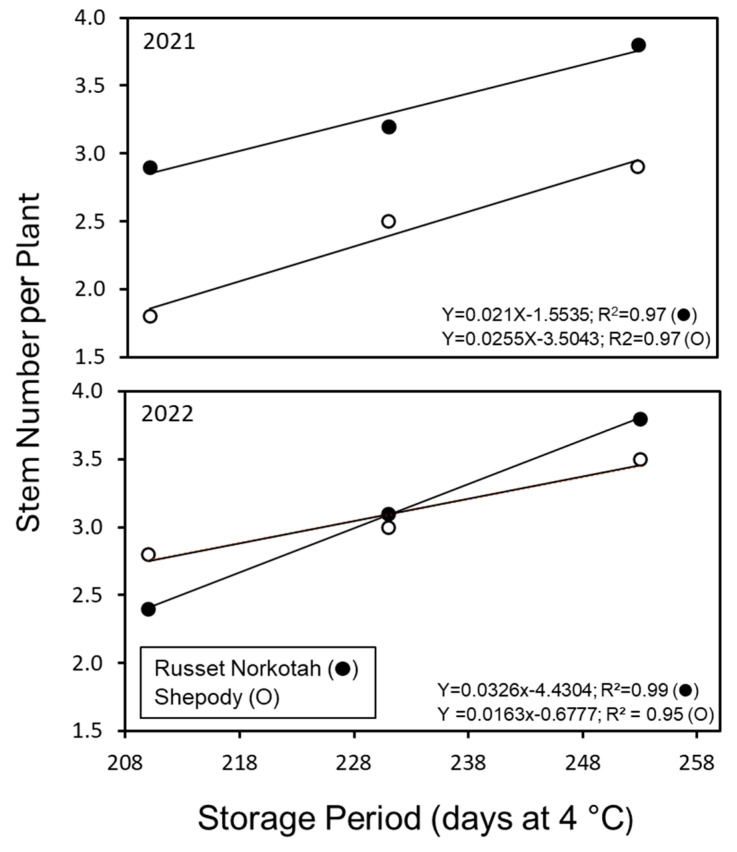
The effect of storage period and planting time the average number of stems per plant (● Russet Norkotah, ○ Shepody). The chronological ages of tubers at planting were 210 (early planting), 231 (mid-planting) and 253 (late planting) days at a constant temperature (4 °C). The stems were counted 60 days after planting. Stem numbers were positively correlated with advancing storage period irrespective of the cultivar or growing year (2021/2022).

The effect of planting time on average stem number plant^−1^ was significant within and across the two growing seasons of 2021 and 2022 (*p* < 0.001; Table 3). The main effect of cultivar on stems numbers plant^−1^ was significant (*p* < 0.001) only during the year 2021. The interaction of planting time with cultivars failed to elicit significant differences in stems plant^−1^ during either of the growing seasons. Irrespective of the cultivar, or the year, the stems plant^−1^ increased linearly with advancing planting time (*p* < 0.001). In 2021, Shepody increased the most stems between planting times (1.1 stems plant^−1^) compared to Russet Norkotah (0.9 stems plant^−1^). Conversely, in 2022, Russet Norkotah had the greatest change in the average number of stems (1.4 stems plant^−1^) while Shepody only had a change of 0.7 stems plant^−1^. The average number of stems also varied by year as demonstrated by the early planted Shepody having a 56% change (2.8 stems plant^−1^) over that of 2021 (1.8 stems plant^−1^). Thus, the increase in stem numbers varied with cultivar, growing period based on the planting time, and the growing year. It is worth noting that the planting time directly altered the storage period (pre-planting). Given the late planted treatments were stored longer and increased the number of stems plant^−1^, this represents another important consideration of the factors impacting stem numbers for both cultivars (Figure 6).

### 3.4. Tuber Number per Plant and Specific Tuber Weight

The effects of planting time and cultivars on the number of tubers per plant were inconsistent between the years 2021 and 2022 (Table 3). In general, tuber numbers declined significantly with the advancing planting time irrespective of cropping year or cultivar. Furthermore, the linear trend for reducing tuber number based on planting time was significant (2021, *p* < 0.001; 2022, *p* < 0.05). In 2022, tuber numbers were not affected in Shepody, but tuber numbers declined with planting time in Russet Norkotah. The main effect of planting time on tuber numbers (average of both cultivars) was significant. Advancing planting time from early to late caused a reduction in the tuber numbers in Russet Norkotah by 48% (9.9 vs. 5.1) in 2021 and 30% (6.9 vs. 4.8) in 2022. Shepody responded with a greater reduction in tuber numbers with advancing planting time (56%; 7.0 vs. 3.1) during 2021. Such a reduction was marginal in 2022 (3%; 5.8 vs. 5.6).

The specific tuber weight (STW) is an index of average tuber size. The effect of cultivar, planting time, and their interaction on STW was significant only during 2021 (Table 3). However, Shepody alone exhibited a significant linear trend for plating time (*p* < 0.001). On average (over planting time) Shepody produced larger STWs (ca. 215 g tuber^−1^) than Russet Norkotah (ca. 119 g tuber^−1^) during 2021. During 2022, STW of Shepody and Russet Norkotah did not differ significantly (ca. 128 g tuber^−1^). Yearly variation in STW were evident. In 2021, STW increased with advancing planting time for Russet Norkotah (114.0 g vs. 121.3 g tuber^−1^) but did not affect STW during 2022 (134.4 g vs. 132.1 g). In both years, late plantings caused a reduction in STW in Shepody (2021, 226.8 g vs. 178.0 g; 2022, 133.8 g vs. 115.1 g).

### 3.5. Total Tuber Yield

Significant differences in total tuber yield in response to planting time, cultivar, and their interaction were observed in the year 2021 (Table 4). However, in the year 2022, significant difference in total yield was limited to planting time (*p* < 0.01) and cultivar (*p* < 0.05), and their interaction did not elicit significant differences in total yield. In general, total tuber yield declined with advancing planting time (planting time linear trend, *p* < 0.01), irrespective of growing season (2021/22) or cultivar, although the magnitude of decline was dependent on the cultivar and the planting period. Early and mid-plantings produced relatively higher yields in the year 2021 (ca. 66% higher than 2022). This observation was most pronounced in Shepody, where total yields in 2021 for early and mid-plantings averaged 75.8 MT ha^−1^, while 2022 averaged 34.4 MT ha^−1^. The reduction in total yield for Russet Norkotah between years was less pronounced, with 2021 producing ca. 52.5 MT ha^−1^ compared to 2022 producing ca. 43.0 MT ha^−1^. When compared within the cropping year, late plantings remained consistent for total yield, regardless of the cultivar and the cropping year. Late plantings reduced total yields when compared to the early and mid-plantings (ca. 68% reduction for 2021/22; Table 4). In 2021, late planted Shepody produced a 27.0 MT ha^−1^ while early and midplanting averaged 75.8 MT ha^−1^. During the same year, late planted Russet Norkotah produced a 30.1 MT ha^−1^ while early and mid-planting averaged 52.5 MT ha^−1^. The same trend was observed in 2022, though diminished. In 2022, late planted Shepody produced a 31.0 MT ha^−1^ compared to the ca. 34.4 MT ha^−1^ for early and mid-plantings and Russet Norkotah produced a 30.9 MT ha^−1^ compared to the ca. 43.0 MT ha^−1^ for early and mid-plantings. Thus, Shepody exhibited greater variation in tuber yield over the two cropping years.

### 3.6. Yield of US No. 1s and US No. 2s

While the cultivar and planting time had significant effect (*p* < 0.01) on the yield of US No. 1s (>113 g/tuber) tubers during both growing seasons (2021/22), their interaction elicited significant difference (*p* < 0.01) only in 2021 (Table 4). The yield of US No. 1s was lower in 2022 than that of 2021 in both cultivars. Tuber yields declined linearly with advancing planting time, in both cultivars irrespective of the year. Shepody produced a greater yield of US No. 1 tubers than Russet Norkotah in 2021 but failed to sustain such productivity in 2022. Thus, Shepody was subjected to a greater yearly variation in the productivity of US No. 1 tubers than that of Russet Norkotah.

Cultivars and planting time affected the proportion of US No 2s (tubers > 113 g) but their interaction was only significant in 2021 (Table 4). In contrast to Russet Norkotah, US No 2s contributed to a greater proportion of total tuber yield in Shepody, irrespective of planting time or year. In the year 2021, the proportion of US No 2 category in Shepody accounted for 15%, 27%, and 10% of the total tuber yield from early and mid- and late plantings, respectively. In 2022, US No 2s accounted for 11%, 16%, and 22% of the total yield of early and mid- and late plantings of Shepody, respectively. Interestingly, US No 2s accounted for greater proportion of the total yield in 2021, the year that experienced higher temperatures during the spring and early summer months. It is important to note that in 2022, Shepody suffered a 44% decline tuber yield over that in 2021 (average over planting time). Thus, US No 2s accounted for a larger proportion of total tuber yield in Shepody than in Russet Norkotah.

### 3.7. Tuber Size Distribution

**Figure 7:**
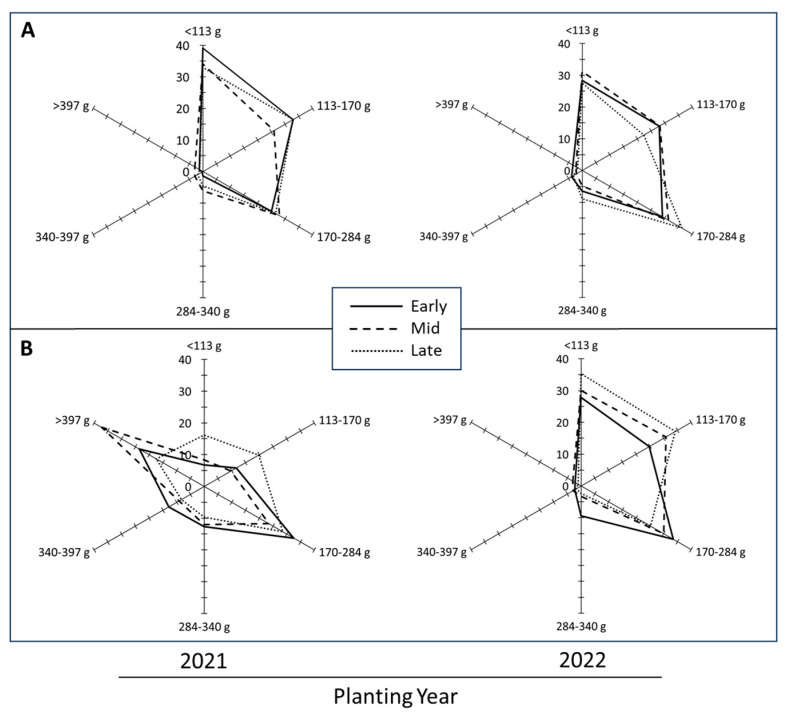
Polygonal diagrams showing the effects of planting time (see Table 1) on the contribution of size categories to the total tuber yield (percent). Each axis represents a specified tuber weight class. The shape of the polygon depicts the effect of planting time on tuber size distribution. Note the differential response of Russet Norkotah (**A**) and Shepody (**B**) and the effect of growing season (2021 vs. 2022) on tuber size profile. In contrast to Shepody, tuber size distributions were less affected by seasonal variation in temperature in Russet Norkotah. The higher temperatures during 2021 increased the proportion of larger tubers (>397 g) specifically in Shepody. Statistical significance of planting time and cultivar on total yield and the contribution of tuber size categories are presented in Table 4, Table 5 and Table 6.

The effect of planting time, cultivar, and their interaction on the yield of tuber size categories differed significantly (*p* < 0.01) during the year 2021 for most of the tuber size categories except for the 113 to 170 g category, where the interaction of planting time and cultivar was non-significant (Table 5). Such differences, however, did not manifest in the year 2022 (Table 6). The relative contribution of different tuber sizes as percentage to the total yield is presented in the polygonal diagrams with each axis representing a specified tuber weight class (Figure 7). The shape of the polygon is a comparative presentation of the overall tuber size distribution, as affected by planting time. The tuber size profile of Russet Norkotah (Figure 7A) was affected to a lesser extent than that of Shepody (Figure 7B) by the planting time or year. During 2021, smaller tubers (<113 g) accounted for a greater proportion of tuber yield of early planted Russet Norkotah (39%) than that of Shepody (7%). With advancing planting time, the relative contribution of this category (<113 g) of tubers declined significantly in Russet Norkotah in both 2021 and 2022 (linear trend, *p* < 0.01 and *p* < 0.05, respectively). Similarly, the size category of 113 to 170 g also decreased with advancing planting time in both cultivars. Shepody produced a greater proportion of larger tubers (>397 g) from early and mid-plantings during 2021 than the late planting and the 2022 crop year. Therefore, delayed plantings reduced the average tuber size in both study years for Shepody (Figure 7B). Interestingly, the mid-planting time had the greatest effect on large tubers (>397 g) in 2021 but not in 2022 (Figure 7; Table 5 and Table 6).

### 3.8. Processing Quality

The effect of planting time, cultivar, and storage period on fry color was examined by measuring photovolt reflectance units (RU) at harvest and after 60 days at 4 °C on the 2022 crop (Table 7). Higher reflectance values are positively correlated with lighter-colored fries and lower reducing sugars [28]. Fry colors immediately after harvest, and averaged over cultivars, had lighter apical (bud) ends (50.2 RU) than the basal (stem) ends (34.1 RU; 16.1 RU difference = 32% decline). A difference between the two ends (apical vs. basal) greater than 9.0 RU are considered ‘non-uniform’ and not suitable for processing. The basal color is specifically referenced, as it is consistently darker than the apical portion and represents the color’s lower limit. At harvest fry color (basal end) was the darkest for the early plantings for both cultivars (storage time = *p* < 0.01). For Russet Norkotah and Shepody early planting fry colors were ca. 27% and 8% darker, respectively, than the mid- and late plantings. After 60 days of tuber storage, the reflectance units (averaged over cultivars) declined significantly (*p* < 0.01) in both apical (42%) and basal (40%) ends, indicating a darker fry color due to the accumulation of reducing sugars at 4 °C (cold-induced sweetening). A similar trend for the effect of planting time on basal end fry color at harvest was observed after the 60 days of storage. Russet Norkotah and Shepody fry colors were ca. 23% and 15% darker, respectively, than the mid- and late plantings. Overall, the main effect of cultivar on fry color was significant (*p* < 0.01). Shepody produced 22% lighter fries than that of Russet Norkotah, though there were no difference for uniformity performance (RUs: Russet Norkotah = 13.2 and Shepody = 14.5, respectively).

### 3.9. Accelerated Aging and Storage Duration Affects Emergence and Stem Numbers

Russet Norkotah and Shepody tubers harvested from the early, mid-, and late plantings were stored at different storage temperatures to develop tubers accumulating 80 DD or 600 DD (see Materials and Methods, Table 8). Planting time had a significant effect on the sprout emergence in the subsequent generation. For example, tubers from early plantings (2021) subjected to 80 DD (control) treatment, had a higher percent emergence than those from the late harvest (Russet Norkotah, 45% vs. 23% and Shepody, 20% vs. 3%; Table 8). Accelerated aging (600 DD) resulted in similar results, although the magnitude of change differed (Russet Norkotah: 40% vs. 27% and Shepody: 22% vs. 11%). Emergence was evaluated at 29 DAP in 2023 due to yearly variation in soil temperature and emergence rates. The trend in emergence from tubers harvested from 2022 (planted in 2023) were similar to that observed for tubers harvested in 2021. Tubers from early plantings and those that accumulated 600 DDs had greater sprout emergence. Russet Norkotah ranged from 58% to 38% (80 DD; early vs. late, respectively) and 76% to 61% (600 DD; early vs. late, respectively). Shepody ranged from 40% to 18% (80 DD; early vs. late, respectively) and 39% to 24% (600 DD; early vs. late, respectively). Irrespective of the year or cultivar, accelerated aging treatments improved sprout emergence marginally in tubers harvested from late plantings.

**Figure 8:**
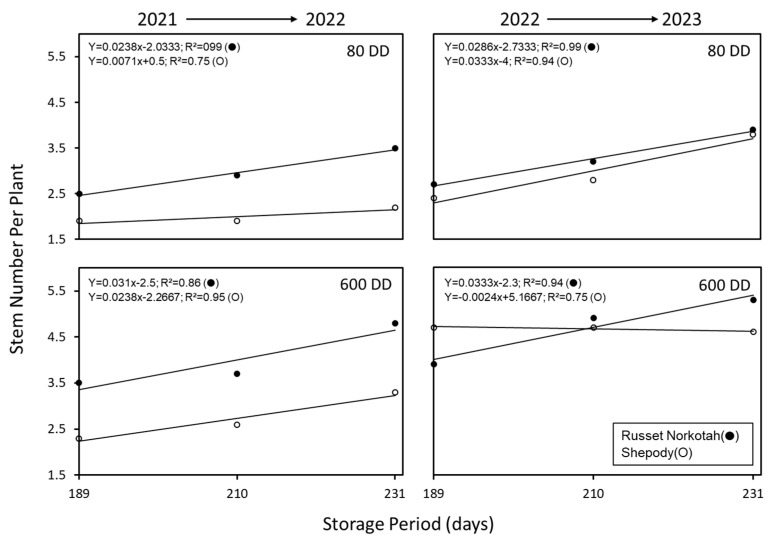
The effect of storage period and temperature on the average number of stems per plant. The tubers of Russet Norkotah and Shepody harvested from early, mid-, and late plantings (Table 1) in the year 2021 and 2022 were subjected to accelerated aging at 12 °C (10 days) or at 32 °C (21 days). Following this aging treatment, the tubers were held at 4 °C until planting in the field. The tubers accumulated 80 degree days at 12 °C (control) and 600 degree days at 32 °C. Tubers were planted in the subsequent years of 2021 and 2022 (2022 and 2023). Stem numbers increased linearly with storage period in controls irrespective of the cultivar or growing year. Both Russet Norkotah and Shepody tubers harvested in 2021 and 2022 responded to accelerated aging (600 DD) with increased stems per plant when planted the following year (2022 and 2023, respectively).

Accelerated aging (600 DD) caused Russet Norkotah to produce a greater number of stems plant^−1^ irrespective of the tuber source (early, mid-, or late planting), year (2022 or 2023), or cultivar, over that of their respective 80 DD controls (Table 8 and Figure 8). The stems plant^−1^ (average over planting time and growing season) increased from 3.1 (80 DD) to 4.4 (600 DD) for Russet Norkotah and 2.5 (80 DD) to 3.7 (600 DD) for Shepody. Thus, in response to accelerated aging, stems plant^−1^ increased by 42% and 48% in Russet Norkotah and Shepody, respectively. The higher percent increase in stems plant^−1^ in Shepody (48%) is a function of lower initial number of stems plant^−1^ in control tubers (2.5) when compared to Russet Norkotah (3.1).

In addition to the effect of accelerated aging on stems plant^−1^, storage duration (chronological time) increased stems plant^−1^ similar to the effect advancing planting time (early, mid, and late) on stem number during the previous production years (see Table 3). During 2021 and 2022, tubers were planted after 210 (early), 231 (mid), or 253 days (late) of storage (Table 1). This difference between the planting periods was 21 to 22 days between plantings and resulted in an increase of ca. 0.58 stems plant^−1^ for Russet Norkotah and 0.45 stems plant^−1^ for Shepody (ca. 0.03 and 0.02 stems plant^−1^ day^−1^, respectively). Interestingly, tubers from the 2021 and 2022 crop years were stored after harvest for a period of 189 to 231 days, which represents 21 days between harvest points (Table 8, Figure 8). These tubers, when planted the subsequent season, had similar changes in stems plant^−1^ based on the storage length with stems plant^−1^ increasing with prolonged storage periods (Table 8). The difference in stems plant^−1^ between growth periods the prior year (early, mid, and late) were ca. 0.55 stems plant^−1^ for Russet Norkotah and 0.43 stems plant^−1^ for Shepody, again ca. 0.03 and 0.02 stems plant^−1^ day^−1^, respectively. This observation demonstrates a consistent effect of chronological age on the average number of stems plant^−1^. Interestingly, tubers with increased age (600 DD) had a similar stems plant^−1^ trend due to chronological age. The Russet Norkotah stems plant^−1^ changed 0.68 stems plant^−1^ and Shepody changed by 0.23 stems plant^−1^ between storage periods (0.03 and 0.01 stems plant^−1^ day^−1^, respectively). This equates to an estimated 20% change in the number of stems plant^−1^ when planting time is altered by 20 days on average for both cultivars. Furthermore, the change in stems plant^−1^, elicited by chronological time, is equivalent and is independent of the physiological status of the tuber. This observation is one factor regulating the stem number ratio for potato tubers.

## 4. Discussion

### 4.1. Temperature and GDDs

Our understanding of the effect of temperature on physiological and molecular aspects of growth and development of the potato is largely derived from studies carried out in controlled environments such as greenhouses or growth chambers [4,9,29,30]. Although the effects of temperature on growth and development are reproducible under controlled environments, its extrapolation to a field-setting presents limitations due to additional environmental factors such a soil heterogeneity, variations in climatic conditions, pests, etc. The magnitude of impact of temperature on crop performance in a field setting is a function of (1) diurnal changes in temperature, (2) the duration and frequency of extremities such as heat waves, (3) modulation of soil temperatures by changes in canopy cover, and (4) growth stages experiencing fluctuations in temperatures. In this study, we examined the effect of natural variations in temperature on growth and productivity of potato in the field setting. The changes in soil and air temperatures were monitored daily during the entire cropping period (134 days) every 15 min. The response of cultivars to changes in average and threshold temperature at different stages of crop growth were examined by adopting the staggered planting technique. In addition, the effect of diurnal fluctuations in soil and air temperatures during tuberization and bulking, differences in the accumulation of growing degree days, maximum/minimum temperatures, and extreme heat events (2021 heat dome) were also considered. The entire experiment was repeated for two years to account for the yearly variations The effect of planting time on growth and productivity was assessed during the growing seasons 2021 and 2022. The effect of planting time on the physiological status of the resultant tubers were assessed during the years that followed 2021 and 2022. Both soil and air temperatures (daily averages and threshold temperatures) were higher in 2021 than in 2022. The temperatures reached a peak 30 days earlier in 2021 than in 2022. The emergence-to-tuberization stage of early plantings (2021) coincided with the peak temperatures (Figure 2 and Figure 3). The total number of air- and soil-GDDs (Figure 5) followed a similar trend of average temperatures, as GDDs were based on average temperatures. The interaction of temperature during different crop growth stages modulating emergence, average number of stems plant^−1^, and yield (total tuber yield, tuber number, and size profile) are discussed below.

### 4.2. Emergence and Stem Numbers

Uniform sprout emergence ensures better crop-stand and tuber yield. Soil temperature plays a key role in emergence and subsequent sprout growth. Optimum soil temperatures for maximum sprout emergence are around 10 to 13 °C at the time of planting. Seed tubers warmed to 10 to 13 °C prior to planting further facilitates emergence. After sprouting, a higher air temperature (20 to 22 °C) favors sprout development [31]. In the present study, sprout emergence rate increased with advancing planting time concomitant with an increase in soil and air temperatures during both years despite a significant yearly variation in soil and air temperatures from emergence-to-tuberization (Figure 2 and Figure 3; Stages I, II). Early planting delayed sprout emergence due to lower soil temperatures irrespective of the year, which increases the risks of soilborne diseases. However, the rate of emergence from early plantings during 2021 was faster than in 2022, likely a consequence of higher soil temperatures during 2021. The average soil temperature during the initial 28 DAP were 16.0 °C and 12.2 °C during 2021 and 2022, respectively (Figure 2 and Figure 3). The emergence from early planted Shepody reached 65% at 28 DAP (2021) in contrast to 0% during 2022 (Table 3). Under similar soil temperature regimes, early planted Russet Norkotah established an 84% emergence (2021), but its emergence in 2022 was similar to Shepody (0%). Thus, the ability to sustain faster emergence is dependent on the cultivar, dormancy status and soil temperatures [32]. Shepody has a longer dormancy, and its emergence is affected more by soil temperatures than Russet Norkotah.

An increase in stem numbers is positively correlated to tuber numbers and negatively correlated to tuber size [33,34]. The number of stems per tuber increases with advancing physiological age (PAGE) in response to a gradual loss in apical dominance [35,36]. Dependable biochemical and/or molecular markers for predicting PAGE prior to sprouting remain elusive. Although stem numbers are dependable markers, the assessment of the PAGE by this method is possible only upon sprouting. Biotic and abiotic factors modulate tuber maturity and thus physiological age [37]. It is postulated that tubers experiencing higher temperatures during maturity (growing degree days) advance into physiologically older tubers in storage. In fact, physiological age can be manipulated by storage temperature and duration of storage [34,38,39]. Our understanding of the effects of the physiological age on stem numbers, tuber set, and tuber size distribution is mainly derived from studies involving manipulation of the physiological age of seed tubers by storage temperature and duration (degree days). Several preharvest factors (crop fertility management programs, soil moisture regimes, and temperature) interact with cultivars to modulate tuber maturity. Such tubers respond differently to the storage temperature affecting their physiological age. In addition, cultivars respond differently to accelerated aging by degree-day accumulation in storage [38]. Therefore, it is unreasonable to draw generalized inferences from the results of such studies across cultivars and growing conditions [40]. The seed tubers used in our study (Russet Norkotah and Shepody) were held at a constant temperature (4 °C) for 210, 231, and 253 days from harvest (chronological age, CAGE) prior to planting in the field (Table 1). This ‘staggered planting’ enabled approached allowed the assessment of the effect of storage period (planting time) and environmental temperatures on average plant stem counts. During the two growing seasons (2021 and 2022), the plants were exposed to soil and air temperatures that were distinctly different (Figure 2 and Figure 3). Despite higher soil and air temperatures from planting to emergence during 2021 than that of 2022, stem numbers increased with storage period irrespective of cultivar or the year (Table 3 and Figure 6). It is important to note that these tubers were procured from the same source and at the same time each year. A short-term exposure (days) of an individual seed-piece to higher soil temperatures at planting (late planting treatment) is unlikely to advance the physiological age to increase the average number of stems per plant. Moreover, lower soil temperatures (early planting, 2022) did not cause a reduction in the number of stems per plant. In fact, early planted Shepody produced 32% more stems during 2022 than that of 2021 (Table 3). Therefore, the increase in stem numbers was mainly a function of storage period (CAGE) and not mediated physiological aging induced by the short-term exposure to increased soil temperatures in 2021. This observation is supported by earlier work by Blauer et al. [39], in which late season aging (via elevated storage temperatures) displayed reduced effectiveness of increasing the stems plant^−1^. Danieli et al. [41] also found an increase in stem numbers with advancing storage period, a consequence of reduced apical dominance.

Manipulation of the physiological age by accelerated aging in storage assumes uniformity in tuber maturity at harvest. Several preharvest conditions (e.g., cropping duration and fertility practices) affect tuber maturity and tubers differing in maturity are morphologically alike. In view of the intrinsic variation in tuber maturity, accelerated aging is unlikely to result in predictable, specific numbers of stem plant^−1^ (even within the same cultivar), though the average trend of increasing stem numbers will likely be observed. In this study, tubers differing in maturity were produced by exposing different stages of crop growth to variations in yearly diurnal soil and air temperatures. Such tubers were subjected to accelerated aging at 12 °C (80 DD) or at 32 °C (600 DD) for a specified period (see Table 8). Following aging treatments, tubers were held at a constant temperature (4 °C) for 189, 201, and 234 days prior to planting the following year, to assess the effect of tuber maturity, accelerated aging, and storage duration on emergence and stem numbers (phenotypic markers of physiological age). The effect of accelerated aging on emergence was inconsistent (Table 8). However, the average number of stems plant^−1^ increased with all aging treatments (cultivar, planting period, and year) by at least 21% to 68%, though there was no discernable trend in the percentage increase in the average number of stems plant^−1^ as impacted by the growth environment nor tuber maturation average temperature (120 to 134 DAP), suggesting that the climatic conditions played a minor role in regulating stems plant^−1^ in the subsequent generation. Interestingly, the average number of stems plant^−1^ increased linearly with storage period (CAGE) irrespective of the cultivar or growing year. Thus, the relative role of CAGE vs. PAGE on the average number of stems is dependent upon the timing and the location (e.g., storage) of the temperature treatments, and suggests CAGE is a primary regulator of stem numbers when considering growing environment while PAGE is critical when considering both the growing and storage environment.

### 4.3. Tuber Count, Size-Distribution, and Yield

It is generally accepted that exposure of potato plants to temperatures above 30 °C day/20 °C night, results in a disproportionate allocation of photosynthates favoring foliage at the cost of tuber growth [7,8,29,42,43,44,45,46]. High temperatures are inhibitory to the transcription of StSP6A, which is involved in tuber initiation and thus tuber yields [29,47,48,49]. The optimum temperature for photosynthesis in potatoes is around 24 °C [43]. However, photosynthesis remains unaffected up to 38 °C and in some cultivars up to 40 °C [42].

Cultivars responded differently to the prevailing air and soil temperatures at different stages of development during 2021 and 2022. For example, early and mid-plantings of 2021 produced a greater number of tubers per plant, higher tuber specific weight (Table 2) and total yield (Table 4) than that of 2022 plantings. Higher temperatures in 2021 likely enhanced photosynthetic ability during tuberization and sustained the development of a greater number of tubers, whereas the lower soil and air temperatures during tuberization in 2022 reduced tuber set. Our results are consistent with a 10-year observation from the Western Regional Trials with Russet Burbank in Parma, Idaho where warmer temperatures during tuber initiation was associated with increased tuber set [50]. Interestingly, this trend did not continue with the late planting period in both 2021 and 2022. The soil and air temperatures were consistently higher for late plantings during both years (2021 and 2022), relative to the early and mid-plantings (Figure 2 and Figure 3). Late planting (2021) caused a reduction in tuber numbers irrespective of the cultivar. However, a similar trend was not observed in late planted Shepody in 2022 (Table 3). It appears that temperature during sprout emergence interacts with temperature during tuberization to modulate tuber yields.

A change in the number of tubers impacts the tuber size distribution and the contribution of different tuber size categories to total yield (Figure 7). Tuber size dictates their suitability for processing into French fries. Very large (>397 g) and very small (<113 g) tubers are less preferred for processing and for ware potatoes (table stock/fresh market). However, these size categories are a part of the total yield and grower financial returns. Russet Norkotah and Shepody responded to higher temperatures (2021) with varying proportions of tuber sizes. While Russet Norkotah produced a greater proportion of smaller (<113 g) tubers, Shepody produced a greater proportion of larger tubers (>397 g). The yield profile of Shepody (Figure 7B) was thus affected to a greater extent by planting time or year than that of Russet Norkotah (Figure 7A). During 2021, smaller tubers (<113 g) accounted for a greater proportion of tuber yield of early planted Russet Norkotah (39%) than that of Shepody (7%). With advancing planting time, the relative contribution of this category (<113 g) of tubers declined marginally in Russet Norkotah. Shepody produced greater proportion of larger tubers (>397 g) from early and mid-plantings during 2021 (Figure 7B). Interestingly, the effect of planting time and cultivar did not affect the contribution of larger tubers to the total yield of either cultivar during the year 2022. A reduction in tuber numbers per plant is generally associated with increased specific tuber weight due to a greater allocation of photosynthates to limited number of tubers. However, the specific tuber weights were significantly lower than that of 2021 (Table 2), suggesting a reduced ability for assimilation and/or translocation of photosynthates. Compared with 2021 results, tuber numbers per plant declined in both cultivars. While a marginal increase in the specific tuber weight (11%) was evident in Russet Norkotah, in Shepody, a decline in tuber numbers was associated with a significant reduction in specific tuber weight (58%). Thus, enhanced ability for photosynthesis, mediated by higher day temperatures during tuberization and lower night (air/soil) temperatures during bulking (Figure 4), most likely contributed toward higher tuber yield in 2021.

In 2022, Russet Norkotah and Shepody lost 22% and 121% yield, respectively, over the average tuber yield of early and mid-plantings in 2021. A reduction in the number of tubers per plant and changes in the specific tuber weight (STW) were factors in the reduced yield. In 2022, the number of tubers per plant (average of early and mid-planting) were 33% and 24% lower (Russet Norkotah and Shepody, respectively) than in 2021. Shepody also had an 80% reduction in STW, but Russet Norkotah had a 10% increase. Unfortunately, the STW increase for Russet Norkotah did not compensate for the loss of tuber numbers, and thus the yield. These changes in tuber numbers and STW affected the tuber size distribution (Figure 7) and can impact crop financial returns. While the percentage of tubers less than 170 g for Russet Norkotah was greater in 2021 (ca. 66% total yield; average early and mid-plantings) than 2022 (ca. 58% total yield). However, the contribution of tubers greater than 170 g was 8% higher in 2022, which accounted for a 12% increase in the STW of Russet Norkotah. Conversely, for Shepody, the contribution of tubers less than 284 g increased in 2022 (ca. 89% total yield; average early and midplantings) over 2021 (ca. 46% total yield; average early and mid-planting). The contribution of tubers greater that 284 g decreased ca. 42% (2022). Interestingly, in 2021, the >397 g category alone accounted for 30% of the total tuber yield (Table 5 and Table 6).

Tuber yields declined with the advancing planting time and the loss in tuber yield was greater in late plantings, irrespective of the cultivar or the year (Table 4). While all plantings reached a 100% crop-stand in due course, the late planting favored faster emergence than the earlier plantings. Despite faster emergence, late plantings produced lower tuber counts and total yields (Table 3). In 2021, late planted Shepody and Russet Norkotah lost 64% and 43% of tuber total yield, respectively, compared with their respective average total yields from the early and mid-plantings. A similar loss in yield was evident in 2022 for the late plantings, although the overall loss in total yield was higher. The early and mid-plantings of 2022 experienced a cooler spring followed by a 30-day delay in the onset of warmer seasonal temperatures than that of 2021 (Figure 2 and Figure 3). These yearly variations in temperature during crop growth stages likely caused differences in tuber yield between the two years.

The impact of temperature on tuber yield depended on its alignment with the crop’s growth stages. Although daily average air and soil temperatures exceeded the optimal recommendations for photosynthesis (30 °C day/20 °C night), their effect on total yield remained positive—provided these high temperatures did not occur during plant establishment (emergence), as seen with late plantings (Figure 3). Additionally, temperatures above the recommended optimum suggest that consistently higher temperatures during tuberization (45–70 DAP) and tuber bulking (71–120 DAP; Figure 3) favor increased tuber yields. A closer examination of daily average temperatures across developmental stages between years (Figure 4) indicates that higher diurnal soil and air temperatures during tuberization, followed by relatively stable lower temperatures (as observed in 2021) during bulking, likely enhance photosynthetic capacity and tuber yield. Conversely, in 2022, tuberization occurred under slightly lower temperatures than in 2021 (average early and mid-planting temperature; Figure 4), but bulking was followed by relatively higher day and night temperatures. Under these conditions, the production and conservation of photosynthates were negatively affected, thus leading to a reduced tuber yield. These year-to-year observations suggest that field conditions with higher tuberization temperatures, followed by relatively lower bulking temperatures enhance photosynthate accumulation in tubers and improve overall yield.

### 4.4. Postharvest Processing Quality

Potato tubers exposed to higher temperatures during bulking and maturity respire at an elevated rate both at harvest and during acclimation to cold storage [24]. Such tubers contain higher amounts of reducing sugars (RS) at harvest and then further accumulate more RS in response to cold temperatures in storage [24,51] in a process known as ‘cold-induced sweetening’ [52]. During frying, RS reacts with amino acids at high temperatures (Maillard reaction), producing darker fries which are considered unacceptable for processing [53]. Fry color, measured by photovolt reflectance units (RUs), serves as an index of RS content, with lower RU values indicating higher RS concentrations and darker fries. Additionally, fry color can serve as an indicator of relative tuber maturity (PAGE).

Although Russet Norkotah is not a processing cultivar and Shepody is unsuited for long-term storage, a fry color assessment was used to evaluate the impact of planting period (early, mid, or late) on RS accumulation at harvest and after storage (4 °C). At harvest, RUs were higher in the apical portion of the tuber (ca. 50 RU; average of planting time and cultivars), indicating lighter fries and relatively lower RS concentrations than in the basal portion (ca. 34 RU; Table 7). This aligns with previous findings that tubers accumulate more RS in the basal portion (stem-end) in response to transient high temperatures during tuber development [54,55]. After 60 days of storage at 4 °C, RUs dropped to 29 (apical) and 17 (basal), reflecting the cold-induced accumulation of RS (average of planting time and cultivars). While the basal RU values at harvest were acceptable for processing (>20 RU) across all planting periods, the difference between the apical and basal tuber portions exceeded nine RU, creating a non-uniform fry color that would cause a rejection of the crop for processing [56]. This non-uniformity was consistent with previous findings in Washington, where production practices not tailored to specific genotypes often result in color deviations [56]. Notably, non-uniformity was more pronounced in earlier plantings, particularly for Russet Norkotah (Table 7).

After 60 days of storage at 4 °C, fry color RUs were unacceptable due to the accumulation of RS, a consequence of Russet Norkotah and Shepody lacking the cold-sweetening resistance trait. Overall, earlier plantings exhibited lower RUs than late plantings for both cultivars in 2022. Despite all plantings undergoing the same duration of growth under identical agronomic practices, fry color data highlight the significant role of temperature in influencing crop maturity (PAGE). Earlier plantings consistently experienced warmer temperatures during late bulking, whereas later plantings were exposed to cooler conditions. These results demonstrate that seasonal temperature variations during tuber development directly impact processing quality by altering RS accumulation patterns. Moreover, these temperature effects persist during longer-term storage (60 days), with later plantings maintaining a lighter fry color compared to earlier plantings.

## 5. Conclusions

Many potato production areas are experiencing temperatures fluctuations and temperatures higher than optimum as a consequence of climate change. The effect of temperature on growth and development of potatoes was examined in a field-setting for two consecutive seasons (2021 and 2022). By adopting staggered plantings, plants differing in developmental stages were subjected to variations in diurnal air and soil temperatures. Sprout emergence, stem numbers, tuber numbers per plant, specific tuber weight, tuber size distribution and total tuber yield were analyzed. In general, the air and soil temperatures during the growing season of 2021 were higher than that of 2022 and exceeded the optimum tuberization temperatures. Contrary to the expectations, higher temperatures (2021) favored earlier emergence, greater number of tubers per plant and higher yields. The stem numbers per plant increased with advancing planting period but did not result in an increase in the number of tubers per plant. Early plantings were not subjected to higher temperatures during emergence in (2021 and 2022) and during tuberization (2022). Late planting exposed the crop to higher annual temperatures during emergence (2021) and tuberization (2022) than that of the early and mid-plantings. Late plantings experienced warmer temperatures during emergence and tuberization and a relatively cooler bulking period (compared to the early and mid-plantings). These conditions significantly decreased total yield, US No. 1 yield, and the number of tubers per plant. Although early and mid-plantings had acceptable yields under high-temperature regimes, tubers bulked and matured in warmer conditions resulting in darker stem-end colored French fries.

The effect of temperature during crop development on seed tuber performance was also examined. Potatoes subjected to temperature variations during development (2021 and 2022) were stored at different temperature regimes to accumulate either 80 or 600 DD and planted in the years that followed 2021 and 2022. The effect on average stem numbers per plant was assessed as a marker of physiological age. The number of stems increased linearly with advancing storage period (chronological age, CAGE) and was unaffected by the in-season temperature variations to which mother plants were subjected during tuber development. While the diurnal fluctuations in temperature did not affect the average number of stems per plant, the storage temperature did affect stem numbers in tubers subjected to accelerated aging (600 DD). Thus, the impact of temperature on the PAGE is a function of timing, duration, and intensity. Adjusting planting time to coincide emergence with modest temperatures, tuberization with warmer temperatures, and bulking with relatively cooler temperatures, has potential for maximizing tuber yields. Furthermore, cooler temperatures during the latter part of bulking and maturation produce tubers of better processing quality. While the effect of temperature during growth is minimal on physiological age, storage temperature and storage duration have a greater bearing on the physiological status thus affecting performance of seed tubers.

## Figures and Tables

**Table 1:** Shifts in the crop developmental stages by staggered planting. Planting was done at three different times (2021/22): Apr 19/18, May 10/09, and June 1/May 31 for early, mid-, and late planting, respectively. The durations of growth stages are based on the previously established chronology specific to the Columbia basin. Seed tubers were stored for the storage period (210–253 days) at 4 °C (95% RH) prior to planting. Irrespective of planting time, the crop was raised for 120 days each crop year and tubers were harvested after a 14-day maturation period under dead vines (134 days in the field).

		Duration of Growth Stages (Days)			
		Emergence	Tuberization	Bulking	Maturity
Seed Storage (days)	Planting	0–45	46–70	71–120	121–134
210	Early	18 Apr–1 Jun	2 Jun–26 Jun	27 Jun–15 Aug	16 Aug–30 Aug
231	Mid	9 May–23 Jun	24 Jun–18 Jul	19 Jul–6 Sep	7 Sept–20 Sept
253	Late	31 May–14 Jul	15 Jul–9 Aug	10 Aug–28 Sep	29 Sept–12 Oct

**Table 2:** Duration and number of hours of day and night during the potato (*Solanum tuberosum* L.) growing season (April–October) used to calculate diurnal variation in air and soil temperatures presented in Figure 4.

	Day		Night		Hours	
Date	AM	PM	PM	AM	Day	Night
21-Apr	6:15	7:45	8:00	6:00	13.50	10.50
1-May	5:15	8:30	8:45	5:00	15.25	8.75
1-Jun	5:00	8:45	9:00	4:45	15.75	8.25
1-Jul	5:15	8:45	9:00	5:00	15.50	8.50
1-Aug	6:00	8:00	8:15	5:45	14.00	10.00
1-Sep	6:45	7:00	7:15	6:30	12.25	11.75
1-Oct	7:15	6:00	6:15	7:00	10.75	13.25

**Table 3:** Effect of planting time and cultivars on emergence, stem number, tuber number, and specific tuber weight (STW) during the growing seasons of 2021 and 2022. Seed tubers used for early, mid-, and late planting had a storage period of 210, 231, and 253 days, respectively (see Table 1). Sprout emergence was recorded at 28 days after planting (DAP) and expressed as a percentage of the total number of seed pieces planted. Stem numbers per plant were counted at 60 DAP. Following harvest (134 days), tuber numbers per plant and individual tuber weights were determined (LSD, least significant difference; LT, linear trend; DEV, deviations from linear trend).

		% Emergence		Stems Plant^−1^		Tubers Plant^−1^		STW (g. Tuber^−1^)	
Cultivar (CV)	Planting Time	2021	2022	2021	2022	2021	2022	2021	2022
Russet Norkotah	Early	84	0	2.9	2.4	9.9	6.9	114.0	134.4
	Mid	97	16	3.2	3.1	8.5	6.9	120.8	127.6
	Late	98	19	3.8	3.8	5.1	4.8	121.3	132.1
Shepody	Early	65	0	1.8	2.8	7.0	5.8	226.8	133.8
	Mid	81	1	2.5	3.0	6.5	5.1	239.8	124.2
	Late	95	14	2.9	3.5	3.1	5.6	178.0	115.1
LSD_0.05_		12	4	0.6	0.6	1.4	1.2	22.7	19.8
CV ^a^		0.01 ^b^	0.001	0.001	ns	0.001	0.1	0.001	ns
Time_LT_		0.001	0.001	0.001	0.001	0.001	0.05	0.05	ns
Time_DEV_		ns	ns	ns	ns	0.01	ns	0.01	ns
CV × Time_LT_		0.1	ns	ns	ns	ns	0.05	0.01	ns
CV × Time_DEV_		ns	0.001	ns	ns	ns	0.05	0.05	ns
RN × Time_LT_		0.05	0.05	0.01	0.001	0.001	0.01	ns	ns
RN × Time_DEV_		ns	0.05	ns	ns	ns	0.05	ns	ns
Shep × Time_LT_		0.001	0.001	0.001	0.05	0.001	ns	0.001	0.1
Shep × Time_DEV_		ns	0.05	ns	ns	0.05	ns	0.001	ns

^a^ Sources of variation. ^b^ Levels of significance (0.1, 0.05, 0.01, 0.001, ns = not significant).

**Table 4:** Effects of planting time and cultivars on tuber yield (total and US No 1s and 2s; metric tons (MT) ha^−1^) during the growing seasons of 2021 and 2022. Note the variation in yield parameters as affected by cultivars and growing seasons (LSD, least significant difference; LT, linear trend; DEV, deviations from linear trend).

		Tuber Yield Categories					
		Total Yield ^a^		US #1 (>113 g)		US #2 (>113 g)	
Cultivar (CV)	Planting Time	2021	2022	2021	2022	2021	2022
Russet Norkotah	Early	54.3	44.2	33.0	31.2	0.1	0.3
	Mid	50.6	41.8	33.4	28.2	0	0.6
	Late	30.1	30.9	19.7	18.8	0.4	3.6
Shepody	Early	76.3	38.0	59.7	23.3	11.5	4.1
	Mid	75.2	30.7	48.5	16.4	20.3	5.0
	Late	27.0	31.0	20.0	13.2	2.6	6.9
LSD_0.05_		11.4	8.1	9.4	7.8	5.6	2.5
CV ^b^		0.001 ^c^	0.05	0.001	0.001	0.001	0.001
Time_LT_		0.001	0.01	0.001	0.001	0.05	0.01
Time_DEV_		0.001	ns	0.05	ns	0.001	ns
CV × Time_LT_		0.01	ns	0.001	ns	0.05	ns
CV × Time_DEV_		0.05	ns	ns	ns	0.001	ns
RN × Time_LT_		0.001	0.01	0.01	0.01	ns	0.01
RN × Time_DEV_		0.1	ns	0.1	ns	ns	ns
Shep × Time_LT_		0.001	0.1	0.001	0.05	0.01	0.05
Shep × Time_DEV_		0.001	ns	0.05	ns	0.001	ns

^a^ MT ha^−1^. Includes US No. 1, 2, undersize (<113 g) and culls. ^b^ Sources of variation. ^c^ Levels of significance (0.1, 0.05, 0.01, 0.001, ns = not significant).

**Table 5:** Effects of planting time and cultivar on the yield of different tuber size categories (metric tons (MT) ha^−1^) during 2021. The percentage contribution of various tuber size categories to the total yield are presented in Figure 7. (LSD, least significant difference; LT, linear trend; DEV, deviations from linear trend).

		Tuber Size Categories ^a^					
Cultivar (CV)	Planting Time	<113 g	113–170 g	170–284 g	284–340 g	340–397 g	>397 g
Russet Norkotah	Early	21.2	17.9	13.6	0.7	0.1	0.7
	Mid	17.2	13.2	14.1	3.1	1.5	1.5
	Late	9.9	9.9	8.0	1.3	0.6	0.4
Shepody	Early	5.1	8.9	24.8	9.7	9.8	18.0
	Mid	6.3	7.3	17.6	9.0	6.9	28.1
	Late	4.4	5.4	7.7	2.6	2.2	4.7
LSD_0.05_		3.8	3.1	4.7	5.8	2.7	7.4
CV ^b^		0.001 ^c^	0.001	0.01	0.001	0.001	0.001
Time_LT_		0.001	0.001	0.001	0.01	0.001	0.05
Time_DEV_		ns	ns	ns	0.01	ns	0.001
CV × Time_LT_		0.001	0.1	0.01	0.001	0.001	0.05
CV × Time_DEV_		ns	ns	ns	ns	ns	0.01
RN × Time_LT_		0.01	0.001	0.05	ns	ns	ns
RN × Time_DEV_		ns	ns	ns	0.1	ns	ns
Shepody × Time_LT_		ns	0.05	0.001	0.001	0.001	0.01
Shepody × Time_DEV_		ns	ns	ns	0.05	ns	0.001

^a^ Units are in MT ha^−1^. ^b^ Sources of variation. ^c^ Levels of significance (0.1, 0.05, 0.01, 0.001, ns = not significant).

**Table 6:** Effects of planting time and cultivar on the yield of different tuber size categories (metric tons (MT) ha^−1^) during 2022. The percentage contribution of various tuber size categories to the total yield are presented in Figure 7. (LSD, least significant difference; LT, linear trend; DEV, deviations from linear trend).

		Tuber Size Categories ^a^					
Cultivar (CV)	Planting Time	<113 g	113–170 g	170–284 g	284–340 g	340–397 g	>397 g
Russet Norkotah	Early	12.6	12.4	12.9	2.9	1.7	1.5
	Mid	13.0	11.8	13.1	2.0	0.9	0.9
	Late	8.5	6.9	11.2	2.7	1.0	0.5
Shepody	Early	10.6	9.4	12.7	3.6	0.9	0.8
	Mid	9.2	9.5	9.2	1.0	0.9	0.9
	Late	10.9	10.6	7.8	0.7	0.6	0.4
LSD_0.05_		3.8	2.7	4.7	2.0	1.3	1.3
CV ^b^		ns	ns	0.1 ^c^	ns	ns	ns
Time_LT_		ns	0.05	0.05	0.05	ns	ns
Time_DEV_		ns	ns	ns	ns	ns	ns
CV × Time_LT_		0.1	0.01	ns	0.05	ns	ns
CV × Time_DEV_		0.1	ns	ns	ns	ns	ns
RN × Time_LT_		0.05	0.001	ns	ns	ns	ns
RN × Time_DEV_		ns	0.1	ns	ns	ns	ns
Shepody × Time_LT_		ns	ns	0.05	0.01	ns	ns
Shepody × Time_DEV_		ns	ns	ns	ns	ns	ns

^a^ Units are in MT ha^−1^. ^b^ Sources of variation. ^c^ Levels of significance (0.1, 0.05, 0.01, 0.001, ns = not significant).

**Table 7:** Effect of cultivar and planting time on the photovolt reflectance of French fries prepared from tubers at harvest (0 day) and after 60 days of storage at 4 °C for the 2022 crop (see Materials and Methods). The difference in reflectance units between the apical (A) and basal (B) portions is indicative of non-uniform fry color. Note: Values 9.0 and above (A-B) represent French fries with non-uniform color. (LSD: Fisher’s least significant differences).

			Photovolt Reflectance Units		
Cultivar (CV)	Planting Time	Storage (d)	Apical	Basal	A–B
Russet Norkotah	Early	0	49.3	23.0	26.3
	Mid		50.2	32.9	17.3
	Late		46.5	30.1	16.4
	Early	60	23.5	14.0	9.5
	Mid		20.5	18.3	2.3
	Late		25.3	17.9	7.4
Shepody	Early	0	50.3	37.4	12.8
	Mid		52.5	40.9	11.6
	Late		52.4	40.4	11.9
	Early	60	29.5	15.8	13.7
	Mid		34.6	18.8	15.7
	Late		39.9	18.4	21.5
LSD_0.05_				3.4	4.7
CV ^a^				0.01 ^b^	ns
Storage Time				0.01	0.01
Planting Time				0.01	ns
CV × Planting Time				0.01	0.01
CV × Planting Time × Storage Time				ns	ns

^a^ Sources of variation. ^b^ Levels of significance (0.1, 0.05, 0.01, 0.001, ns = not significant).

**Table 8:** The effect of planting time, cultivar, and storage temperature on emergence and stem numbers per plant (index physiological age). Tubers harvested during 2021 and 2022 from early, mid-, and late plantings (tuber source) were stored at 12 °C or 32 °C for 10 or 21 days (d) to induce accelerated aging (AA) and later stored for the specified periods at 4 °C (95% + RH) before planting (April 28) in the years following 2021 and 2022. Percentage emergence and average stem number per plant were recorded at row closure (approximately 60 DAP) and are presented below. (LSD: Fisher’s least significant differences).

		AA (d)		Storage (d)		% Emergence				Stems Plant^−1^	
Cultivar (CV)	Tuber Source	12 °C	32 °C	4 °C	DD	2022 (36 DAP)	2022 (43 DAP)	2023 (29 DAP)	2023 (36 DAP)	2022	2023
Russet Norkotah	Early	10	-	231	80	45	94	58	93	3.5	3.9
		-	21	231	600	40	91	76	99	4.8	5.3
	Mid	10	-	210	80	42	90	60	98	2.9	3.2
		-	21	210	600	43	96	66	98	3.7	4.9
	Late	10	-	189	80	23	76	38	98	2.5	2.7
		-	21	189	600	27	67	61	99	3.5	3.9
Shepody	Early	10	-	231	80	20	87	40	93	2.2	3.8
		-	21	231	600	22	76	39	94	3.3	4.6
	Mid	10	-	210	80	8	77	31	95	1.9	2.8
		-	21	210	600	15	78	32	99	2.6	4.7
	Late	10	-	189	80	3	80	18	94	1.9	2.4
		-	21	189	600	11	88	24	94	2.3	4.7
LSD_0.05_						17	18	23	7	0.5	0.6
CV ^a^						0.001 ^b^	ns	0.001	0.05	0.01	ns
Planting Time						0.01	0.1	0.01	ns	0.01	0.01
DD						ns	ns	0.1	0.1	0.01	0.01
CV × Planting Time						ns	0.01	ns	ns	ns	0.1
CV × DD						ns	ns	ns	ns	ns	ns
Planting Time × DD						ns	ns	ns	ns	ns	0.1
CV × Planting Time × DD						ns	ns	ns	ns	ns	0.05

^a^ Sources of variation. ^b^ Levels of significance (0.1, 0.05, 0.01, 0.001, ns = not significant).

## Data Availability

The original contributions presented in this study are included in the article. Further inquiries can be directed to the corresponding author(s).
